# Treatment burden, adherence, and quality of life in children with daily GH treatment in France

**DOI:** 10.1530/EC-22-0464

**Published:** 2023-03-28

**Authors:** Régis Coutant, Maithé Tauber, Béatrice Demaret, Robin Henocque, Yves Brault, François Montestruc, Olivier Chassany, Michel Polak, the QOLITHOR Study Group

**Affiliations:** 1 Department of Pediatric Endocrinology and Diabetology, Reference Center for Rare Pituiatry Diseases, University Hospital of Angers, Angers, France; 2 Reference Center for the Prader-Willi syndrome and other rare obesities with feeding disorders (PRADORT), Children Hospital, CHU Toulouse, Toulouse, France; 3 Pediatric team of the Clinical Investigation Center 9302/INSERM, Hospital of Children, Toulouse, France; 4 Institut Toulousain des Maladies Infectieuses et Inflammatoires (Infinity), INSERM UMR1291 - CNRS UMR5051 - Université Toulouse III, Toulouse, France; 5 GRANDIR - French Growth Disorders Association, Asnières-sur-Seine, France; 6 Pfizer France, Paris France; 7 eXYSTAT, Malakoff, France; 8 Health Economics Clinical Trial Unit (URC-ECO), Hospital of Hotel-Dieu, AP-HP, Paris, France; 9 Patient-Reported Outcomes Unit (PROQOL), UMR 1123, University Paris Cité, INSERM, Paris, France; 10 Hôpital Universitaire Necker Enfants Malades, Pediatric Endocrinology, Gynecology and Diabetology, Imagine Institute, INSERM U1163, Cochin Institute, INSERM U1016, Centre de référence des pathologies endocriniennes rares de la croissance et du développement, Université de Paris Cité, Paris, France

**Keywords:** growth hormone deficiency, patient-reported outcomes, quality of life, recombinant growth hormone, treatment burden

## Abstract

**Objective:**

The objective of this study was to describe in a real-life setting the treatment burden and adherence and quality of life (QOL) of children treated with daily injections of growth hormone and their relationship with treatment duration.

**Design:**

This non-interventional, multicenter, cross-sectional French study involved children aged 3–17 years treated with daily growth hormone injections.

**Methods:**

Based on a recent validated dyad questionnaire, the mean overall life interference total score (100 = most interference) was described, with treatment adherence and QOL, using the Quality of Life of Short Stature Youth questionnaire (100 = best). All analyses were performed according to treatment duration prior to inclusion.

**Results:**

Among the 275/277 analyzed children, 166 (60.4%) had only growth hormone deficiency (GHD). In the GHD group, the mean age was 11.7 ± 3.2 years; median treatment duration was 3.3 years (interquartile range 1.8–6.4). The mean overall life interference total score was 27.7 ± 20.7 (95% CI (24.2; 31.2)), with non-significant correlation with treatment duration (*P* = 0.1925). Treatment adherence was good (95.0% of children reported receiving >80% of planned injections over the last month); it slightly decreased with treatment duration (*P* = 0.0364). Children’s overall QOL was good (81.5 ± 16.6 and 77.6 ± 18.7 according to children and parents, respectively), but subscores of the coping and treatment impact domains were <50. Similar results were observed in all patients independently of the condition requiring treatment.

**Conclusions:**

This real-life French cohort confirms the treatment burden of daily growth hormone injections, as previously reported in an interventional study.

## Introduction

Growth hormone deficiency (GHD) results in abnormal linear growth in children (1, 2), associated with potential early severe morbidity (psychosocial problems and episodes of hypoglycemia). In addition, persistency of GHD into adulthood is associated with increased risk of cardiovascular morbidity and mortality.

Recombinant human GH (rhGH) replacement therapy has been proven to be safe and effective to enable children with short stature to achieve normal height, with early improvement of psychosocial problems (3, 4) and improvement in quality of life (QOL) (5, 6). In addition, rhGH injections are well tolerated (7, 8) even if adherence to treatment was shown as suboptimal (9). The majority of currently available rhGH products require daily subcutaneous injections. However, the burden of long-term daily administration can cause a reduction in adherence (10) and thus limit the therapeutic utility of existing formulations (11).

It was then hypothesized that switching to less frequent injections could lead to more convenience and treatment adherence, as previously shown in other conditions (12). Based on positive efficacy and safety outcomes from a phase III trial (13, 14), somatrogon, a long-acting rhGH for once-weekly subcutaneous administration, was approved in the European Union in February 2022 (15) for the treatment of children and adolescents from 3 years of age with growth disturbance due to insufficient secretion of GH. In addition, a 24-week phase III study (NCT03831880) assessed the perception of treatment burden in weekly GH (somatrogon) vs daily GH (genotropin) injections in children with GHD, with a crossover at 12 weeks. Since treatment burden of the rhGH injection is often a shared experience between patient and caregiver, it was assessed using a Dyad Clinical Outcomes Assessment (DCOA) questionnaire specifically developed and validated prior to this trial to measure rhGH injection treatment burden, with a simultaneous assessment using a parent–child dyad (Life Interference Questionnaire for Growth Hormone Deficiency (LIQ-GHD)) (16). First results showed that treatment with somatrogon administered once-weekly improved the mean overall life interference total score (primary outcome) after 12 weeks of treatment compared to somatropin administered once-daily (mean respective overall life interference total scores: 8.63 and 24.13; difference between mean scores: –15.49; 95% CI: (–19.71, –11.27); *P* < 0.0001) (17).

French available data on treatment burden of daily GH injections are scare, in particular regarding long-term QOL and life interference of treated children and caregivers. In addition, such real-life data are becoming increasingly important and now recommended by French, European, and US Health Authorities to measure patient perceptions and improve quality of care in routine clinical practice (18, 19, 20, 21). In this context, the real-world French QOLITHOR study aimed to describe the treatment burden and adherence as well as the QOL of children treated with rhGH (seven devices available in 2019 in France for once-daily injections). In addition, this study was designed to assess the extent to which these outcomes are associated with the duration of rhGH treatment together with other factors related to demographics and/or treatment.

## Material and methods

### Study design

QOLITHOR is a non-interventional, multicenter, cross-sectional study conducted in 11 hospitals that belong to the French network for rare disorders as well as endocrinologist liberal practice centers throughout France. Eligible patients were children aged between 3 and 17 years, who received daily rhGH injections for at least 4 weeks to treat GHD only or GH insufficiency as part of multiple pituitary hormone deficiencies as well as other conditions (small for gestational age (SGA), Prader–Willi syndrome (PWS), Turner syndrome, or chronic renal failure). Pediatric patients on hormonal replacement therapy for other hypothalamic pituitary axis hormonal deficiencies and/or diabetes insipidus should be on an optimized and stable treatment regimen for at least 3 months prior to inclusion. Children with cancer history, psychosocial dwarfism, diabetes mellitus, human immunodeficiency virus infection, immunodeficiency syndrome, tuberculosis, or other acute or chronic medical or psychiatric condition were excluded from the study. In order to ensure homogeneity according to rhGH treatment duration, participant centers were asked to enroll eligible children within three patient groups according to their rhGH treatment duration prior to inclusion (<2, (2–5), and ≥5 years). In addition, inclusions were closely monitored to achieve a limited number of patients receiving rhGH for a condition other than GHD only and numbers of included patients per age group sufficient for consistent analysis.

Written consent has been obtained from each patient or subject after full explanation of the purpose and nature of all procedures used. The study was conducted in compliance with the ethical standards of the 1964 Helsinki declaration and its later amendments, the deontology guidelines and Good Epidemiology Practices, and the French regulation on non-interventional studies. The study protocol was approved on March 5, 2021, by the independent Ethics Committee of ‘*Ile de France III’* (reference number: 2020-A02952-37).

### Data collected

The following data were collected by physicians at the inclusion visit: patient and disease characteristics (demographics, disease history, and auxological data), current rhGH treatment, and concomitant medication.

**Figure 1:**
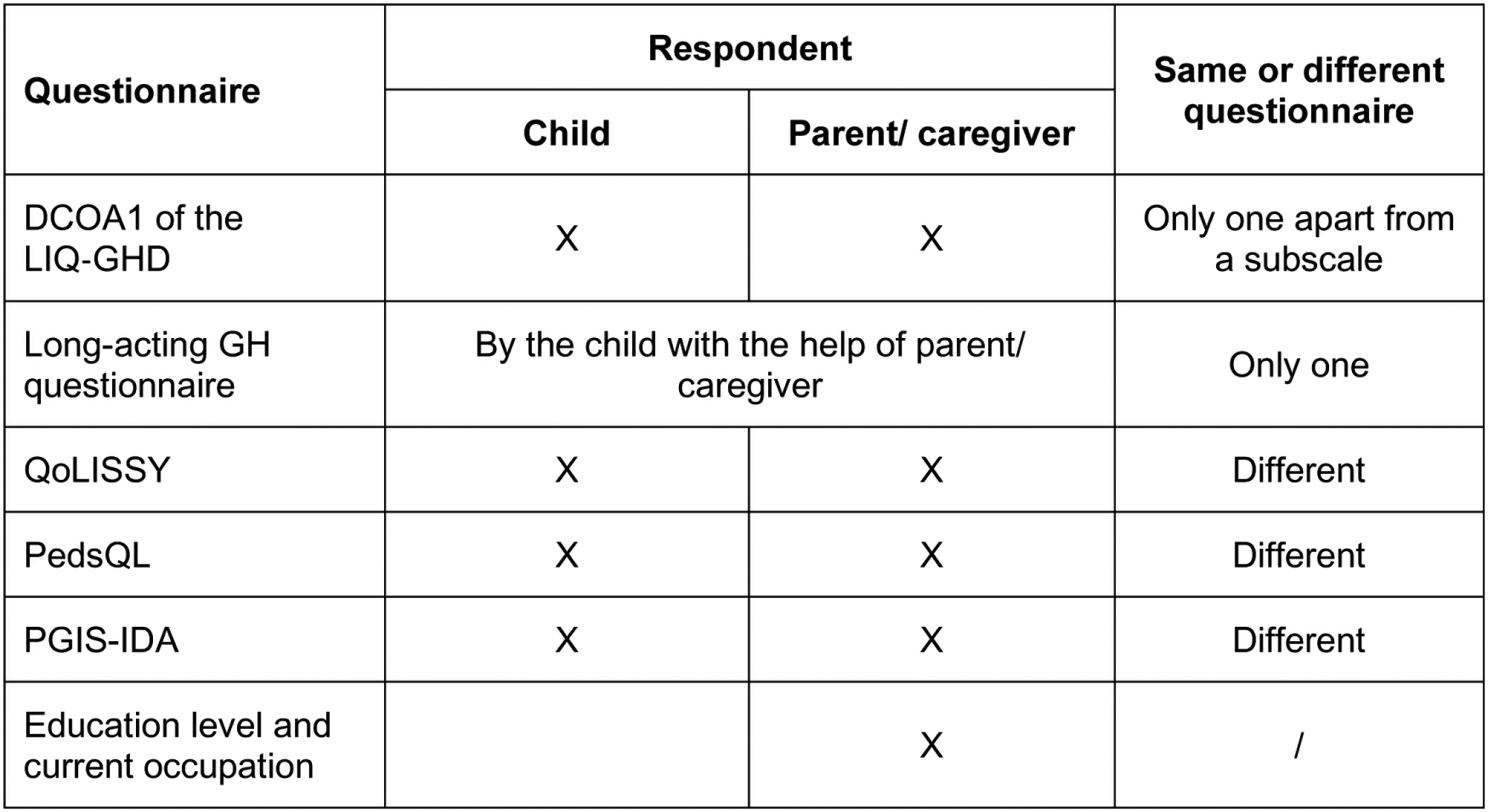
Self-reported questionnaires completed by children and parents/caregivers. DCOA, Dyad Clinical Outcomes Assessment; GH, growth hormone; LIQ-GHD, Life Interference Questionnaire for Growth Hormone Deficiency; PedsQL, Pediatric Quality of Life Inventory; PGIS-IDA, Patient Global Impression Severity – Impact on Daily Activities; QoLISSY, Quality of Life of Short Stature Youth. The DCOA1 of the LIQ-GHD covers life interference due to regimen, missed injections, pen ease of use, regimen convenience, benefit/satisfaction/willingness to continue treatment, and injection-related signs/symptoms.

Children and parents/caregivers completed six self-reported questionnaires (Fig. 1) on rhGH treatment burden (DCOA1) (16), QOL (Quality of Life of Short Stature Youth (QoLISSY) (22) and Pediatric Quality of Life Inventory (PedsQL) (23)), perceived impact on daily activities (Patient Global Impression Severity – Impact on Daily Activities, PGIS-IDA), and education level and occupation of parent/caregiver. DCOA1, QoLISSY, PedsQL, and PGIS-IDA are questionnaires validated in the French language.

The DCOA1 is a part of the LIQ-GHD which was previously used in the NCT03831880 trial to assess treatment burden in GHD children receiving rhGH treatment (16). It contains 22 questions covering the following domains: life interference due to regimen, missed injections, pen ease of use, regimen convenience, benefit/satisfaction/willingness to continue treatment, and injection-related signs/symptoms. The QoLISSY, aiming to measure QOL in short stature youth, assesses QOL in three core domains (physical, social, and emotional), three additional domains to measure predictors of QOL (coping, beliefs, and treatment), and two supplementary domains referring to the parent’s worries about their child’s future (future) and the impact of the child’s condition on the parents’ well-being (effects on parents). The PedsQL, a generic tool aiming to measure children’s QOL, can be completed by children, with versions available for children aged 5–7, 8–12, and 13–18. Parent-rated versions are available for children aged 2–4, 5–7, 8–12, and 13–18. The questionnaire includes four generic core scales (physical, emotional, social, and school). The PGIS-IDA assesses the severity of the impact of the disease on daily activities of parents/caregivers, children according to the point of view of parents/caregivers, and children themselves, using 7-point Likert scales.

### Statistical methods

Descriptive statistical analysis was performed in the population of all the eligible children (with GHD only and other or multiple deficiencies such as SGA, Turner syndrome, or PWS) and in the group of children with only GHD, overall and according to the treatment duration prior to inclusion (<2, (2–5), and ≥5 years). DCOA1, QoLISSY, PedsQL, and PGIS-IDA were analyzed according to the authors’ instructions, and the total scores were converted on 0–100 scores for ease of interpretation. On this basis, a score of 100 corresponded to the most interference with patient daily life for the overall life interference total score, the best QOL for the QOLISSY and PedsQL scores, the strongest preference for weekly rhGH injections for the long-acting questionnaire, and the strongest severity of the disease impact on daily activities for the PGIS-IDA.

The primary criterion of interest was based on the life interference, a part of the DCOA1 completed by children and parents. The primary outcome of the study was the relationship between the overall life interference total score and the duration of treatment in children of the GHD group. A total of 255 eligible pediatric patients were expected in this study: 164 children with only GHD (GHD group) and 91 children affected by another condition. Considering 164 patients in the GHD group, the correlation test (two-sided Fisher's *z* test) of *ρ* = 0 (*α* = 0.05 two-sided) for the level of the overall life interference total score according to the GH treatment duration (analyzed as a continuous variable) had 90% power to detect a correlation coefficient *ρ* of at least 0.25.

The relationship between the overall life interference total score and the rhGH treatment duration (continuous variable) was analyzed using a linear regression model. The associated correlation test (two-sided Fisher's *z* test) and the Pearson correlation coefficients were provided with associated 95% two-sided CI.

Linear regression models were also used to analyze the relationship between the other scores of the DCOA1 questionnaires, the QoLISSY score, the PedsQL score, and the treatment duration (in years). For the relationship between the treatment adherence as reported over the last month preceding the participation in the study by children and parents/caregivers (≤80% (6–28 missed injections) or >80% (0–5 missed injections) over the last 28-day period) and the treatment duration (<2, (2–5), and ≥5 years), a chi-square test for trend in proportions based on a binary logistic model was used.

To search for prognostic factors of treatment burden, adherence, and QOL, multivariate linear regression models were used. Univariate analyses were first performed using pertinent variables, after checking conditions of application of the model (the list of the tested variables is provided in Supplementary Data, see section on supplementary materials given at the end of this article). After selection of variables associated with the dependent variable with a *P* value ≤0.2, checks of multicollinearity between variables were performed; then a backward selection of variables with a significance level of 0.05 was applied.

## Results

### Patient and disease characteristics

**Figure 2:**
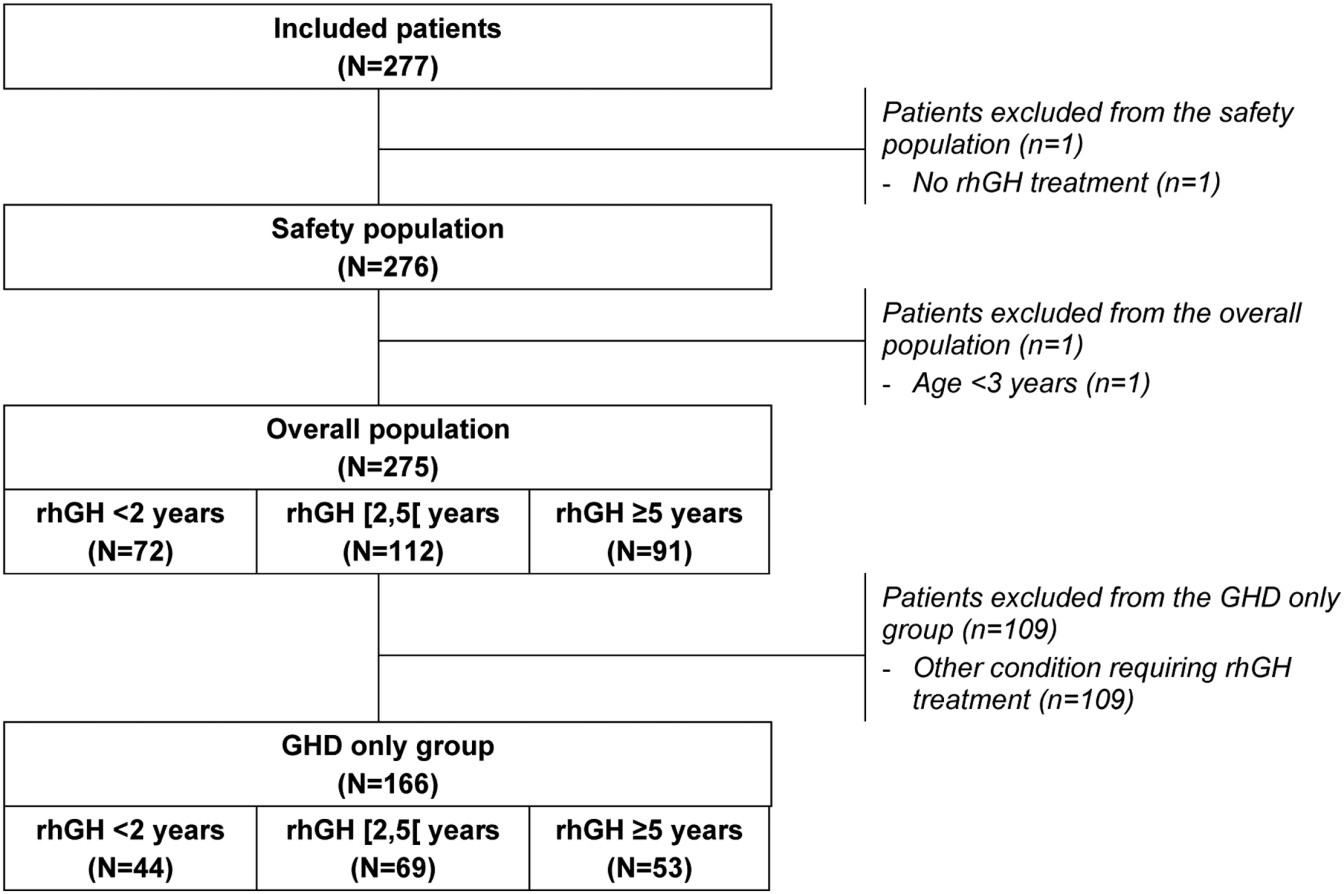
Study populations. GHD, growth hormone deficiency; rhGH, recombinant human growth hormone.

Study populations are presented in Fig. 2. Among the 277 included children, 275 were retained in the overall population for analysis after the exclusion of two patients who did not meet the study selection criteria. Among these 275 patients, 166 (60.4%) were included in the GHD group, 87 (31.6%) were born SGA, 11 (4.0%) suffered from Turner syndrome, and 11 (4.0%) from PWS.

Patient and disease characteristics are detailed in Table 1. The 275 analyzed children of the overall population were aged 11.5 ± 3.3 years at inclusion and were more often male (60.7%). Compared to normal growth curves, children had a smaller height at inclusion (–1.2 ± 0.9 s.d.), but this difference tended to decrease when the duration of the rhGH treatment increased.

**Table 1:** Patient and disease characteristics.

Parameters	Overall population				GHD group			
	rhGH <2 years (*n* = 72)	rhGH 2–5 years (*n* = 112)	rhGH ≥5 years (*n* = 91)	Total (*n* = 275)	rhGH <2 years (*n* = 44)	rhGH 2–5 years (*n* = 69)	rhGH ≥5 years (*n* = 53)	Total (*n* = 166)
Indication of rhGH treatment, *n* (%)								
GHD	44 (61.1%)	69 (61.6%)	53 (58.2%)	166 (60.4%)	44 (100.0%)	69 (100.0%)	53 (100.0%)	166 (100.0%)
Small for gestational age	25 (34.7%)	37 (33.0%)	25 (27.5%)	87 (31.6%)				
Turner syndrome	3 (4.2%)	1 (0.9%)	7 (7.7%)	11 (4.0%)				
Prader–Willi syndrome	0 (0.0%)	5 (4.5%)	6 (6.6%)	11 (4.0%)				
Etiology of deficiency, *n* (%)								
Congenital					43 (97.7%)	67 (97.1%)	48 (94.1%)	158 (96.3%)
Idiopathic					34 (79.1%)	56 (83.6%)	29 (60.4%)	119 (75.3%)
Other^a^					9 (20.9%)	11 (16.4%)	19 (39.6%)	39 (24.7%)
Not applicable					0 (0.0%)	0 (0.0%)	0 (0.0%)	0 (0.0%)
Acquired					1 (2.3%)	2 (2.9%)	3 (5.9%)	6 (3.7%)
GHD duration (years), median (IQR)					1.6 (0.9–2.0)	3.7 (2.9–4.7)	8.3 (7.0–10.9)	4.0 (2.2–7.1)
Duration of rhGH treatment (years), median (IQR)	1.0 (0.5–1.6)	3.3 (2.6–4.0)	7.5 (6.3–9.9)	3.5 (1.9–6.3)	1.1 (0.5–1.6)	3.1 (2.5–3.9)	7.8 (6.5–10.5)	3.3 (1.8–6.4)
rhGH dose (mg/kg/day), mean (s.d.)	0.041 (0.012)	0.043 (0.014)	0.039 (0.016)	0.041 (0.014)	0.037 (0.010)	0.041 (0.011)	0.036 (0.011)	0.038 (0.011)
Severity of deficiency, *n* (%)								
Partial					37 (84.1%)	54 (78.3%)	34 (66.7%)	125 (76.2%)
Severe					6 (13.6%)	13 (18.8%)	17 (33.3%)	36 (22.0%)
Unknown					1 (2.3%)	1 (1.4%)	0 (0.0%)	2 (1.2%)
Not applicable					0 (0.0%)	1 (1.4%)	0 (0.0%)	1 (0.6%)
Male sex, *n* (%)	44 (61.1%)	65 (58.0%)	58 (63.7%)	167 (60.7%)	29 (65.9%)	48 (69.6%)	39 (73.6%)	116 (69.9%)
Age (years), mean (s.d.)	10.1 (3.4)	11.2 (3.4)	13.1 (2.3)	11.5 (3.3)	10.3 (3.4)	11.4 (3.3)	13.4 (2.3)	11.7 (3.2)
Height s.d. compared to general population (AFPA), mean (s.d.)	–1.8 (0.8)	–1.2 (0.7)	–0.7 (0.8)	–1.2 (0.9)	–1.7 (0.9)	–1.1 (0.7)	–0.4 (0.7)	–1.0 (0.9)
BMI compared to general population (AFPA), *n* (%)								
Leanness	18 (25%)	20 (18%)	14 (15%)	52 (19%)	13 (30%)	14 (20%)	7 (13%)	34 (20%)
Normal BMI	47 (65%)	84 (75%)	66 (73%)	197 (72%)	24 (55%)	50 (72%)	40 (75%)	114 (69%)
Overweight/obesity	7 (10%)	8 (7%)	11 (12%)	26 (9%)	7 (16%)	5 (7%)	6 (11%)	18 (11%)
Ongoing rhGH treatment, *n* (%)								
Norditropin FlexPro®	32 (44.4%)	41 (36.6%)	26 (28.6%)	99 (36.0%)	20 (45.5%)	26 (37.7%)	19 (35.8%)	65 (39.2%)
Saizen®	22 (30.6%)	19 (17.0%)	20 (22.0%)	61 (22.2%)	14 (31.8%)	15 (21.7%)	11 (20.8%)	40 (24.1%)
Omnitrope®	6 (8.3%)	19 (17.0%)	7 (7.7%)	32 (11.6%)	3 (6.8%)	11 (15.9%)	4 (7.5%)	18 (10.8%)
Other	12 (16.7%)	33 (29.5%)	38 (41.8%)	83 (30.2%)	7 (15.9%)	17 (24.6%)	19 (35.8%)	43 (25.9%)
Time between GHD diagnosis and treatment onset (months), median (IQR)					3.2 (1.8–5.0)	3.6 (2.0–8.8)	3.5 (1.5–6.0)	3.4 (1.9–7.1)
Concomitant aromatase inhibitors, GnRH analog alternative hormone therapy or other injection treatments, *n* (%)	10 (13.9%)	10 (8.9%)	23 (25.3%)	43 (15.6%)	6 (13.6%)	8 (11.6%)	18 (34.0%)	32 (19.3%)
Current occupation of the answering parent/caregiver, *n* (%)								
Professional activity	59 (96.7%)	79 (92.9%)	66 (88.0%)	204 (92.3%)	36 (97.3%)	53 (98.1%)	42 (91.3%)	131 (95.6%)
No professional activity	1 (1.6%)	3 (3.5%)	5 (6.7%)	9 (4.1%)	0 (0.0%)	1 (1.9%)	3 (6.5%)	4 (2.9%)
Did not wish to answer	1 (1.6%)	3 (3.5%)	4 (5.3%)	8 (3.6%)	1 (2.7%)	0 (0.0%)	1 (2.2%)	2 (1.6%)

Missing data (overall population with rhGH <2, (2–5, ≥5 years, total; GHD group with rhGH <2, (2–5), ≥5 years, total): etiology of GHD (0, 0, 2, and 2); severity of deficiency (0, 0, 2, and 2); time between GHD diagnosis and treatment onset (0, 0, 2, and 2); current occupation of the answering parent/caregiver (11, 27, 16, and 54; 7, 15, 7, and 29).

^a^Related to the pituitary gland in 31 patients.

AFPA, Association Française de Pédiatrie Ambulatoire (French Association of Ambulatory Pediatrics); BMI, body mass index; GHD, growth hormone deficiency; GnRH, gonadotrophin-releasing hormone; IQR, interquartile range; rhGH, recombinant human growth hormone.

At inclusion, the median treatment duration of children was 3.5 years (interquartile range: 1.9–6.3) (mean duration: 4.3 ± 3.2 years; <2 years: 26.2%, 2–5 years: 40.7%, ≥5 years: 33.1%). At this time, Norditropin FlexPro® was the most frequent treatment used by children.

Concomitant aromatase inhibitors, gonadotropin-releasing hormone analogs, alternative hormone therapy, or other injection treatments (prescribed in 15.6% of children of the overall population) included thyroxine in 23/275 patients (8.4% of the cases).

### Treatment burden

#### Overall life interference

Among the analyzed children, 225/275 (81.8%) of the overall population completed the life interference questionnaire, including 138/166 (83.1%) of the GHD group (primary criterion), 68/87 patients (78.2%) with SGA, 10/11 (90.9%) with Turner syndrome, and 9/11 (81.8%) with PWS.

The total mean overall life interference score in the GHD group (primary analysis) was 27.7 ± 20.7 (*n* = 138) (Table 2). It was 27.1 ± 18.3 in patients with SGA, and as follows for the smaller groups: 20.7 ± 16.6 in patients with Turner syndrome and 50.0 ± 20.7 in patients with PWS (Table 3).

**Table 2:** Treatment burden of children, using the overall life interference total score.

	rhGH <2 years (*n* = 44)	rhGH (2–5) years (*n* = 69)	rhGH ≥5 years (*n* = 53)	GHD-only group (*n* = 166)
*N*	38	54	46	138
Overall life interference total score:				
Mean (s.d.)	31.4 (20.5)	28.4 (20.1)	23.8 (21.2)	27.7 (20.7)
*95%* CI	(24.6; 38.1)	(22.9; 33.9)	(17.5; 30.1)	(24.2; 31.2)
Correlation with rhGH treatment duration				
Pearson coefficient (95% CI)				–0.111(–0.273; 0.057)
*P*-value				0.1925

GHD, growth hormone deficiency; rhGH, recombinant human growth hormone.

**Table 3:** Treatment burden, treatment adherence, and quality of life of children born small for gestational age (SGA), with Turner syndrome (TS), and with Prader–Willi syndrome (PWS).

	SGA (*n* = 87)	TS (*n* = 11)	PWS (*n* = 11)
Overall life interference total score	*N* = 68	*N* = 10	*N* = 9
Mean (s.d.)	27.1 (18.3)	20.7 (16.6)	50.0 (20.7)
*95%* CI	(22.7; 31.5)	(8.9; 32.6)	(34.1; 65.9)
Number of missed rhGH injections over the last 28 days	*N* = 71	*N* = 10	*N* = 9
Mean (s.d.)	2.5 (4.7)	1.9 (4.6)	4.4 (9.2)
>80% of planned injections done (according to respondents), *n* (%)	64 (90.1%)	9 (90.0%)	7 (77.8%)
QoLISSY score			
Children, mean (s.d.)	*N* = 69	*N* = 9	*N* = 9
	73.1 (17.8)	65.7 (19.2)	84.1 (15.0)
Caregivers, mean (s.d.)	*N* = 66	*N* = 9	*N* = 9
	72.3 (19.5)	65.9 (20.6)	87.7 (9.1)

QoLISSY, Quality of Life of Short Stature Youth; rhGH, recombinant human growth hormone.

In the GHD group, this mean score tended to decrease (less interference) when the treatment duration increased (31.4 ± 20.5 (<2 years), 28.4 ± 20.1 ((2–5) years), 23.8 ± 21.2 (≥5 years)), and no significant correlation with the rhGH treatment duration was observed (Pearson coefficient: *r*= –-0.11, 95% CI (–0.27; 0.06); *P* = 0.1925 – univariate regression analysis). Nevertheless, using multivariate analysis, treatment duration was shown as a prognostic factor of less interference with the daily life of children (interference decrease: –1.13, 95% CI; (–2.21; –0.06) per year of treatment, *P* = 0.0383), as well as seven rhGH injections per week (least square means (95% CI): 36.01 (30.65; 41.37) for 6 days a week vs 28.53 (22.60; 34.46) for seven injections a week; *P* = 0.0365). In contrast, deficiency severity seemed to be a prognostic factor (27.03 (22.76; 31.30) for partial vs 37.52 (29.70; 45.33) for severe, *P* = 0.0217). The device used for rhGH treatment was also a prognostic factor of interference on patient daily life (Supplementary Table 1).

**Figure 3:**
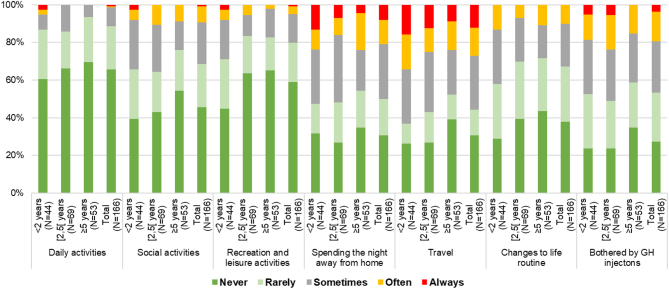
Detailed results from the life interference questionnaire according to the rhGH treatment duration (GHD-only group). GHD, growth hormone deficiency; rhGH, recombinant human growth hormone.

According to the different items of the life interference questionnaire, rhGH injections interfered the most with ‘spending the night away from home’ and ‘travel’ (often or always in 20.7% and 27.1% of the cases, respectively) (Fig. 3).

No correlation was also observed between the mean overall life interference total score and the rhGH treatment duration in the overall population (*r* = 0.01, 95% CI (–0.12; 0.14), *P* = 0.8747). Using multivariate analysis, two independent factors were significantly associated to this score: seven injections (compared to six injections) per week of rhGH were less likely to interfere, and the device used for rhGH treatment was also a prognostic factor of interference on patient daily life (Supplementary Table 2). In univariate analysis, the indication of rhGH treatment (GHD only vs other or multiple deficiencies) was not associated with the total overall life interference score (*P* = 0.7143).

#### Impact of the disease severity on daily activities

Using the PGIS-IDA in the GHD group, the concordance of assessments by children and caregivers was strong between caregivers for themselves and for their children (kappa coefficient: 0.61, 95% CI (0.51; 0.71)), moderate between caregivers for children and children for themselves (0.47 (0.37; 0.58)), and slight between caregivers for themselves and children for themselves (0.371 (0.27; 0.47)).

### Quality of life

**Figure 4:**
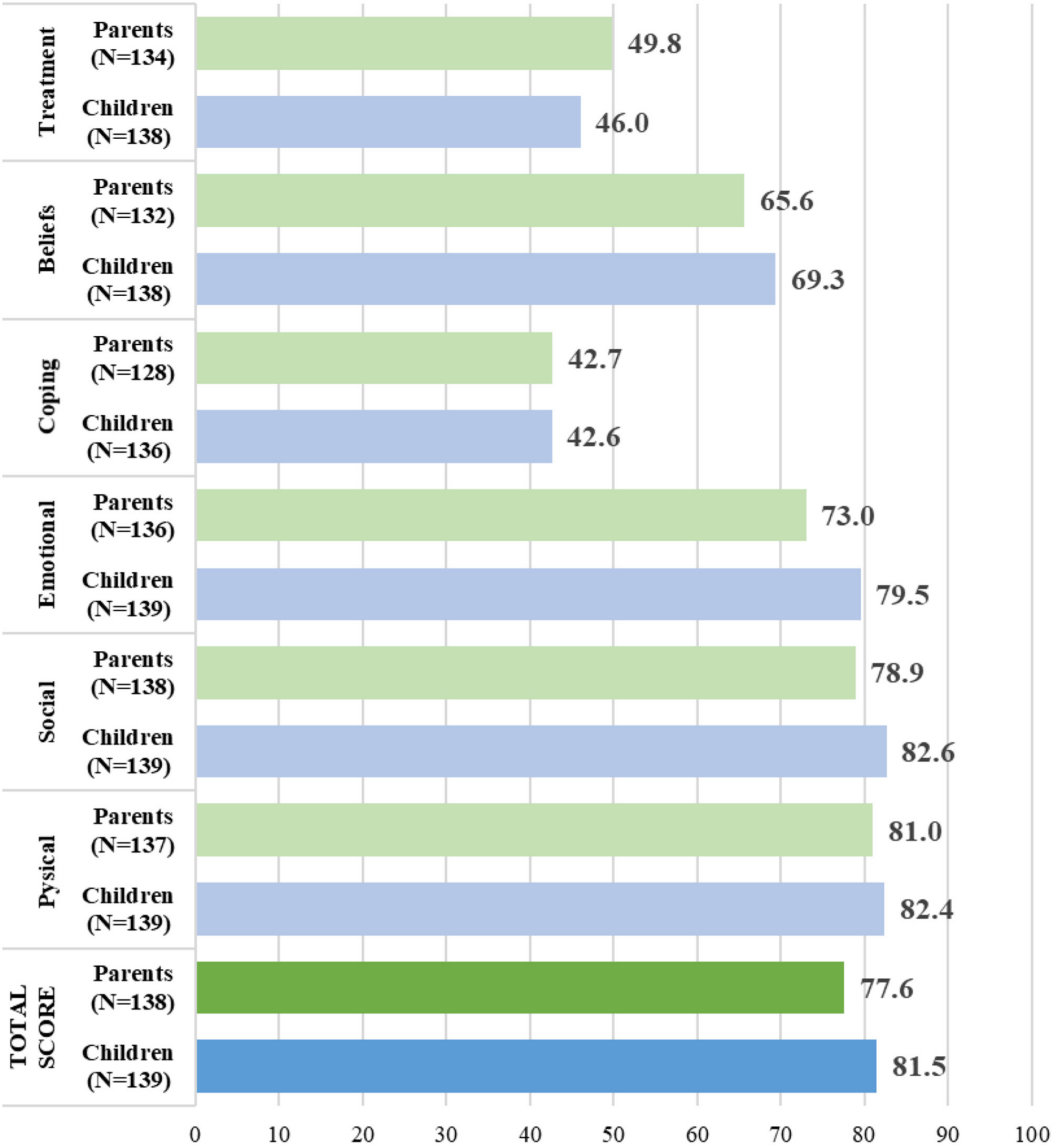
Mean QoLISSY total scores based on children’s and parent’s assessments (GHD only group). GHD, growth hormone deficiency; QoLISSY, Quality of Life of Short Stature Youth.

Using the QoLISSY, a good QOL of children of the GHD group was observed according to children and parents/caregivers (respective mean scores: 81.5 ± 16.6 and 77.6 ± 18.7 on a 0–100 scale where 100 = best QOL) (Fig. 4). For patients with SGA, Turner syndrome, and PWS, similar mean scores between children and caregivers were found: 73.1 ± 17.8 and 72.3 ± 19.5, 65.7 ± 19.2 and 65.9 ± 20.6, and 84.1 ± 15.0 and 87.7 ± 9.1 (Table 3).

In the GHD group, the lowest mean scores were observed for the coping domain (children: 42.6 ± 22.2; parents: 42.7 ± 21.8) and the domain associated with the rhGH treatment (46.0 ± 17.2 and 49.8 ± 18.5, respectively). Both total scores increased with the rhGH treatment duration in the GHD group (*r* = 0.26, 95% CI (0.10; 0.41), *P* = 0.0017 and *r*= 0 .28 (0.11; 0.42), *P* = 0.0009, respectively) and in the overall population (*P* = 0.0005 and 0.0023, respectively). Using multivariate analysis, a high daily dose of rhGH was more likely to lead to a poor children QOL according to children themselves and their parents/caregivers in the GHD group (QOL decrease: –276.51, 95% CI (–510.45; –42.57), *P* = 0.0209 and –300.82 (–586.03; –15.61), *P* = 0.0389, respectively), while a higher height s.d. compared to general population was more likely to lead to a good QOL (QOL increase: 6.72 (3.77; 9.67), *P* < 0.0001 and 5.40 (1.91; 8.89), *P* = 0.0027, respectively) (Supplementary Tables 3 and 4). The same two independent prognostic factors of the children QOL were observed in the overall population (Supplementary Tables 5 and 6). The indication of rhGH treatment (GHD only vs other or multiple deficiencies) was not found as an independent factor associated to the QoLISSY score.

Using the PedsQL, a good QOL was also observed for children of the GHD-only group (mean scores around 80 according to children for themselves and parents for their children, whatever the patient group age was), but no significant correlations with the treatment duration were shown in both populations (*P* = 0.5177 and *P* = 0.3784, respectively).

### Missed rhGH injections

The number of missed injections reported by children and parents/caregivers over the last 28 days of treatment led to a mean score of 1.7 ± 2.2 (0: no missed injections; 28: all injections missed) in patients of the GHD group. On this basis, a high treatment adherence was estimated in 95% of them (>80% of planned injections were done according to respondents). The score for missed injections significantly increased with the duration of rhGH treatment in the GHD group (*r* = 0.24, 95% CI: (0.08; 0.39), *P* = 0.0033) and in the overall population (0.14 (0.01; 0.26), *P* = 0.0364).

In other groups of patients, the mean numbers of missed injections over the last 28 days were 2.5 ± 4.7 for SGA, 1.9 ± 4.6 for Turner syndrome, and 4.4 ± 9.2 for PWS (Table 3), with a high treatment adherence whatever their condition was (in 90%, 90%, and 78% of patients, respectively).

### Factors related to daily rhGH injections impacting patient daily life

Ranking nine factors that may impact children’s daily life of the GHD group (from 1, the most important to 9, the less important), daily injections were reported as the most impacting factor (mean quotation out of 9: 3.0 ± 2.6) compared to others (subcutaneous injections, pain during injections, going away for the weekend or on holidays, visiting or sleeping at friends’ house in the evening, medication storage in refrigerator or not, medical visits, reconstitution steps, and dose adjustment).

## Discussion

Treatment with daily rhGH injections has been shown to promote linear growth in children with GHD until their adult height target has been achieved, lead to few adverse events, and improve their QOL (3, 4, 5, 6, 7, 8). However, treatment adherence to daily injection regimens remains suboptimal, and non-compliance could compromise height velocity in children (9, 10, 11, 24).

After first results from a crossover phase III trial which showed that treatment burden of weekly injections of somatrogon was lower than that of somatropin administered once daily in children with GHD (NCT03831880 trial) (17), the real-world cross-sectional French QOLITHOR study, conducted in 275 pediatric patients including 166 children with GHD, provides complementary information on patient experience as recommended by French, European, and US Health Authorities (18, 19, 20, 21).

Using the recently developed, validated dyad LIQ-GHD questionnaire (16) to assess rhGH treatment burden, the interference of daily injections with the daily life of children with GHD was similar in both studies (mean overall life interference total scores: 27.7 in QOLITHOR vs 24.13 after a 12-week treatment period in the NCT03831880 trial). These results are in favor of the relevance and the consistency of the questionnaire on life interference due to regimen to assess treatment burden in children with GHD.

No correlation between the mean overall life interference total score and the duration of rhGH treatment was observed. Nevertheless, using multivariate analysis, treatment duration was shown as a prognostic factor of less interference with the daily life of children. The interpretation of these results remains difficult as the lack of current therapeutic alternative may probably lead to an adaptation process, despite the fact that the ongoing treatment of children was not optimal. In addition, the efficacy of current rhGH treatments over time may tend to minimize the treatment burden.

Our real-life data showed a good adherence to daily injections of rhGH (according to 95.0% of children and parents, more than 80% of planned injections over the last 28-day period were done) which was consistent with findings from an Italian cross-sectional study conducted in 1007 children (24.4% missed at least one injection during a typical week) (25). However, these self-reported rates were based on respondents’ own declarations, which may lead to an overestimation of treatment adherence. That being said, our study confirmed in a real-life setting that adherence to rhGH injections significantly decreased according to the treatment duration, even if the increase in missed injections was limited. This result was consistent with previous findings (26, 27, 28). Since a significant correlation was reported between the treatment adherence and the growth velocity of children (27, 28), our results tend to highlight the need for a better treatment adherence over time to achieve a better therapeutic efficacy.

The QOLITHOR study showed a high level of QOL in children under daily rhGH injections, using the QoLISSY questionnaire specifically developed for pediatric patients with short stature. QOL was better when assessment was performed by children compared to parents’ evaluation (81.5 vs 77.6), as previously observed (29). For children and parents, child height compared to the general population was found as an independent prognostic factor of a good QOL, which suggests that the possible constraints related to daily injections may be outweighed by the efficacy of their rhGH treatment over time. This result was consistent with previous 1-year findings having showed that height s.d. score gains were positively correlated with QoLISSY self-report score gains (*P* = 0.001) and QoLISSY parent-report gains (*P* = 0.00001) (30). In addition, it should be noted that the QoLISSY domain related to rhGH treatment showed a stronger impact on the QOL of children, with a mean score below 50/100 which is still in line with the impact of daily injections on children life. Coping difficulties were also observed in treated children.

In addition, once-daily injections were assessed by children as the most important treatment-related parameter that may impact their daily life, compared to eight other factors (mean rank out of 9: 3.0). This result is consistent with data from a previous discrete choice experiment study having showed that patients prefer a less frequent injection regimen for treating GHD (31). Our mean overall life interference total score related to daily injections of somatropin was consistent with the one found in the NCT03831880 trial (27.7 and 24.13), and three times higher than the mean score related to weekly injections of somatrogon (8.63, *P* < 0.0001 between groups) (17). As reported in other conditions, a reduced treatment burden may result in improvement treatment adherence (12).

Our study has several limitations. Considering the real-life design of QOLITHOR study, medical assessments were not always available in patients’ medical files. In addition, self-reported questionnaires were not always completed and returned by children and parents. However, the number of missed questionnaires was limited. For analysis, the overall life interference total score (primary criterion of interest) could be calculated in 138 patients of the 166 children with GHD (83.1%). This high completion rate for a non-interventional study highlights the high engagement from caregivers and children.

In conclusion, the real-world French QOLITHOR study confirms the burden associated with daily injections of rhGH treatment in children with GHD, as previously shown in an interventional study.

## Supplementary Material

Supplementary Material

Supplementary Data

## Declaration of interest

RC has received honoraria as scientific advisor or for lectures from Pfizer, Novo-Nordisk, Merck-Serono, Lilly, and Sandoz; MT has received reimbursement of participation fees for scientific meetings, and continuing medical education events as well as lecture honoraria and funding for research projects from Millendo, Pfizer, Novo Nordisk, and Merck Serono; BD has received honoraria for lectures from Ipsen, Merck-Serono, Novo Nordisk, Pfizer, and Sandoz; RH and YB are employees of Pfizer; FM is an employee of eXYSTAT (statistical analysis of the study data); OC has received honoraria from Pfizer; MP has received research support form Pfizer, Novo Nordisk, Ipsen, Sandoz, Merck-Serono, Lilly, and Sanofi, National French grants (public funding, PHRC and ANR), and participated in advisory boards for Increlex (Ipsen), GNAP (Novo Nordisk), and KIGS France (Pfizer).

## Funding

This work was supported by Pfizer France, Paris, France.

## Author contribution statement

RC, MT, and MP: conceptualization, methodology, investigation, validation, and writing – review and editing; BD and OC: conceptualization, methodology, validation, and writing – review and editing; RH: conceptualization, methodology, validation, writing – review and editing, supervision, and funding acquisition; YB and FM: conceptualization, methodology, formal analysis, validation, and writing – review and editing.
