# Supplementary material for: Treatment burden, adherence, and quality of life in children with daily GH treatment in France
Source: Endocr Connect. 2023 Mar 28;12(4):e220464. doi: 10.1530/EC-22-0464 (PMC10083659; doi:10.1530/EC-22-0464)
Supplement: Supplementary Material [file supplementary_material.pdf]

QOLITHOR study group

- Prof. Pascal BARAT, Hôpital Pédiatrique, Bordeaux, France
- Prof. Régis COUTANT, Dr. Natacha BOUHOURS-NOUET, Dr. Aurélie DONZEAU, Dr. Jessica AMSELLEM JAGER, Dr. Stéphanie ROULEAU, and Dr. LEVAILLANT, CHU d'Angers, Angers, France
- Dr. Isabelle FLETCHNER, Centre Médical Spécialisé de l'Enfant et de l'Adolescent, Paris, France
- Dr. Maxime GERARD, Paris, France
- Dr. Christine LEFEVRE, CHU de Lille, Hôpital Jeanne de Flandre, Lille, France
- Prof. Agnès LINGLART and Dr. Cécile THOMAS-TEINTURIER, Hôpital du Kremlin-Bicêtre, Le Kremlin-Bicêtre, France
- Prof. Marc NICOLINO, Hôpital Femme-Mère-Enfant, Bron, France
- Prof. Michel POLAK, Dr. Isabelle FLETCHNER, Dr. Graziella PINTO, Dr. Dinane SAMARA-BOUSTANI, Dr. Athanasia STOUPA, and Dr. Caroline THALASSINOS, AP-HP, Hôpital Necker Enfants Malades, Paris, France
- Prof. Rachel REYNAUD, Dr. Delphine BERNOUX, Dr. Emeline MARQUANT, and Dr Sarah CASTETS, Hôpitaux Publics, Marseille, France
- Prof. Sylvie ROSSIGNOL, Dr. Marie MANSILA, and Dr François BREZIN, Hôpital de Hautepierre, Strasbourg, France
- Prof. Maithé TAUBER and Dr. Béatrice JOURET, CHU de Toulouse, Hôpitaux Mère et Enfant, Toulouse, France
