# Supplementary material for: Treatment burden, adherence, and quality of life in children with daily GH treatment in France
Source: Endocr Connect. 2023 Mar 28;12(4):e220464. doi: 10.1530/EC-22-0464 (PMC10083659; doi:10.1530/EC-22-0464)
Supplement: Supplementary Data [file supplementary_table_1.pdf]

SUPPLEMENTARY DATA

Search for predictive factors of treatment burden (overall Life Interference total score), and quality of life (QOLISSY score).

Baseline variables tested in all univariate analysis:

- Patient age (years)
- Gender (female, male)
- Height SD compared to the general population
- Weight compared to the general population (<P10, [P10-P25[, [P25-P75], >P75)
- Body mass index (leanness, normal, overweight/ obesity)
- Tanner stage (1, 2, 3, 4, 5)
- Etiology of deficiency (acquired, congenital)
- Deficiency severity (partial, severe)
- Daily dose of rhGH treatment (mg/kg/day)
- Duration of the rhHG treatment (years)
- Injections of rhGH done by the patient (never, yes)
- Frequency of rhGH injections (1 injection 6 days a week, 1 injection 7 days a week)
- Current rhGH treatment (Norditropin FlexPro®, Saizen®, Omnitrope®, other)

In addition, in the univariate analyses performed in the overall population, the indication of the treatment (GHD, other condition) was also tested.

Table 1: Prognostic factors of the overall Life Interference total score - GHD group (N=166)

Explanatory variables*	Univariate Analyses			Multivariate Analysis			
	Number of patients	Least-Square (LS) Means [95% CI]	p value	Number of patients	LS Means [95% CI]	Effect estimate [95% CI]	p value**
**Age** (years)	138		0.0803				
**Body mass index (BMI)**			0.1817				
Leanness	29	21.43 [13.88; 28.97]					
Normal BMI	94	29.29 [25.10; 33.48]					
Overweight/ obesity	15	30.00 [19.51; 40.49]					
**Tanner stage**			0.0440				
1	53	32.75 [27.25; 38.25]					
2	19	17.48 [8.29; 26.67]					
3	23	29.81 [21.46; 38.16]					
4	27	22.62 [14.91; 30.33]					
5	15	27.86 [17.52; 38.20]					
**Deficiency severity**			0.0977				0.0217
Partial	106	26.31 [22.32; 30.30]		106	27.03 [22.76 ; 31.30]	NA (Reference)	
Severe	28	33.67 [25.91; 41.44]		28	37.52 [29.70 ; 45.33]	10.4859 [1.557 ; 19.4149]	
**Duration of hGH treatment (years)**	138		0.1921	134		-1.13 [-2.21 ; -0.06]	0.0383
**Frequency of rhGH injection**s			0.0898				0.0365
1 injection 6 days a week	82	30.18 [25.70; 34.66]		81	36.01 [30.65 ; 41.37]	NA (Reference)	
1 injection 7 days a week	56	24.11 [18.69; 29.53]		53	28.53 [22.60 ; 34.46]	-7.4795 [-14.4828 ; -0.4761]	
**Current rhGH medication**			0.0052				0.0082
SAIZEN®	35	33.37 [26.71; 40.03]		35	35.44 [28.02 ; 42.86]	11.9248 [3.4436 ; 20.406]	
NORDITROPINE FlexPro®	54	20.83 [15.47; 26.19]		53	23.51 [17.36 ; 29.67]	NA (Reference)	
OMNITROPE®	16	37.72 [27.87; 47.57]		16	39.60 [29.54 ; 49.65]	16.0834 [5.0014 ; 27.1655]	
Other	33	28.14 [21.28; 35.00]		30	30.55 [23.33 ; 37.77]	7.0365 [-2.1621 ; 16.2351]	

* Only variables with p value <0.2 are presented for univariate analyses

** Only variables with p value <0.05 are presented for multivariate analysis

Table 2: Prognostic factors of the overall Life Interference total score – Overall population (N=275)

Explanatory variables*)	Univariate Analyses			Multivariate Analysis			
	Number of patients	Least-Square (LS) Means [95% CI]	p value	Number of patients	LS Means [95% CI]	Effect estimate [95% CI]	p value**
**Body mass index (BMI)**			0.1644				
Leanness	41	24.74 [18.53; 30.95]					
Normal BMI	162	28.04 [24.92; 31.17]					
Overweight/ obesity	22	34.90 [26.43; 43.38]					
**Height SD compared to the general population (AFPA)**			0.1962				
<-2 SD	36	24.11 [17.47; 30.75]					
≥2 SD	189	28.87 [25.98; 31.77]					
**Weight position relative to general population percentiles (AFPA)**			0.0652				
< P10	65	25.82 [20.92; 30.73]					
[P10; P25[	66	25.92 [21.05; 30.79]					
[P25; P75]	76	29.37 [24.84; 33.90]					
> P75	18	39.09 [29.77; 48.40]					
**Tanner stage**			0.0449				
1	86	31.19 [26.94; 35.44]					
2	32	21.21 [14.23; 28.18]					
3	39	31.96 [25.65; 38.27]					
4	37	22.78 [16.30; 29.26]					
5	30	27.74 [20.54; 34.94]					
**Deficiency severity**			0.1138				
Partial	118	26.36 [22.63; 30.10]					
Severe	29	33.13 [25.60; 40.66]					
**Frequency of rhGH injection**s			0.0222				0.0128
1 injection 6 days a week	137	30.58 [27.20; 33.96]		129	31.85 [28.25 ; 35.46]	NA (Reference)	
1 injection 7 days a week	88	24.27 [20.06; 28.48]		85	24.90 [20.52 ; 29.27]	-6.9572 [-12.4191 ; -1.4954]	
**Current rhGH medication**			0.0163				0.0215
SAIZEN®	50	30.57 [25.02; 36.12]		46	28.87 [23.03 ; 34.70]	6.8699 [-0.3697 ; 14.1095]	

Explanatory variables*)	Univariate Analyses			Multivariate Analysis			
	Number of patients	Least-Square (LS) Means [95% CI]	p value	Number of patients	LS Means [95% CI]	Effect estimate [95% CI]	p value**
NORDITROPINE FlexPro®	84	22.58 [18.29; 26.86]		79	22.00 [17.60 ; 26.40]	NA (Reference)	
OMNITROPE®	27	32.28 [24.72; 39.83]		27	31.05 [23.52 ; 38.57]	9.0467 [0.3561 ; 17.7373]	
Other	64	31.70 [26.79; 36.60]		62	31.59 [26.61 ; 36.57]	9.5897 [2.9702 ; 16.2092]	
**Participating center**			0.0246				
1001	22	28.41 [20.10 ; 36.71]					
1002	11	28.25 [16.50 ; 39.99]					
1003	10	46.43 [34.11 ; 58.75]					
1004	38	22.18 [15.86 ; 28.50]					
1005	21	24.83 [16.33 ; 33.33]					
1007	23	34.47 [26.35 ; 42.60]					
1009	33	31.06 [24.28 ; 37.84]					
1010	27	31.35 [23.85 ; 38.85]					
1011	23	24.07 [15.95 ; 32.19]					
1012	16	18.97 [9.23 ; 28.71]					
1013	1	39.29 [0.33 ; 78.24]					

* Only variables with p value <0.2 are presented for univariate analyses

** Only variables with p value <0.05 are presented for multivariate analysis

Table 3: Prognostic factors of the QoLISSY score for children – GHD group (N=166)

Explanatory variables*	Univariate Analyses			Multivariate Analysis		
	**Number of patients**	**Least-Square (LS) Means [95% CI]**	**p value**	**Number of patients**	**Effect estimate [95% CI]**	**p value****
**Height SD compared to the general population**	139		<.0001	138	6.72 [3.77 ; 9.67]	<.0001
**Weight position relative to general population percentiles (AFPA)**			0.0306			
< P10	38	76.60 [71.38 ; 81.81]				
[P10; P25[	10	93.51 [83.34 ; 103.67]				
[P25; P75]	37	81.56 [76.28 ; 86.84]				
> P75	54	82.62 [78.24 ; 86.99]				
**Duration of hGH treatment (years)**	139		0.0018			
**Daily dose of rhGH** (kg/mg/day)	138		0.0020	138	-276.51 [-510.45 ; -42.57]	0.0209

* Only variables with p value <0.2 are presented for univariate analyses

** Only variables with p value <0.05 are presented for multivariate analysis

Table 4: Prognostic factors of the QoLISSY score for parents/ caregivers – GHD group (N=166)

Explanatory variables*	Univariate Analyses			Multivariate Analysis		
	Number of patients	Least-Square (LS) Means [95% CI]	p value	Number of patients	Effect estimate [95% CI]	p value**
**Height SD compared to the general population (AFPA)**	138		0.0006	137	5.40 [1.91 ; 8.89]	0.0027
**Duration of hGH treatment (years)**	138		0.0010			
**Daily dose of rhGH** (kg/mg/day)	137		0.0080	137	-300.82 [-586.03 ; -15.61]	0.0389

* Only variables with p value <0.2 are presented for univariate analyses

** Only variables with p value <0.05 are presented for multivariate analysis

Table 5: Prognostic factors of the QoLISSY score for children – Overall population (N=275)

Explanatory variables*	Univariate Analyses			Multivariate Analysis		
	Number of patients	Least-Square (LS) Means [95% CI]	p value	Number of patients	Effect estimate [95% CI]	p value**
**Gender**			0.0078			
Female	89	74.55 [70.94 ; 78.17]]				
Male	137	80.88 [77.97 ; 83.80]				
**Height SD compared to the general population (AFPA)**	226		<.0001	226	8.31 [5.91 ; 10.70]	<.0001
**Weight position relative to general population percentiles (AFPA)**			0.0235			
< P10	66	74.71 [70.51 ; 78.90]				
> P75	17	87.56 [79.29 ; 95.83]				
[P10; P25[	68	77.01 [72.88 ; 81.15]				
[P25; P75]	75	80.81 [76.87 ; 84.74]				
**Indication group**			0.0008			
GHD	139	81.47 [78.61 ; 84.34]				
Other condition	87	73.47 [69.84 ; 77.09]				
**Duration of hGH treatment (years)**	226		0.0005			
**Daily dose of rhGH** (kg/mg/day)	225		0.0006			
**Participating center**			0.0179			
1001	23	84.99 [77.97 ; 92.02]				
1002	11	81.41 [71.25 ; 91.57]				
1003	10	75.12 [64.46 ; 85.77]				
1004	37	83.52 [77.98 ; 89.05]				
1005	21	65.68 [58.33 ; 73.03]				
1007	24	75.62 [68.74 ; 82.50]				
1009	34	80.81 [75.03 ; 86.59]				
1010	26	77.34 [70.73 ; 83.94]				
1011	24	75.35 [68.47 ; 82.23]				
1012	15	77.95 [69.25 ; 86.65]				
1013	1	94.79 [61.10 ; 128.48]				

* Only variables with p value <0.2 are presented for univariate analyses

** Only variables with p value <0.05 are presented for multivariate analysis

Table 6: Prognostic factors of the QoLISSY score for parents/ caregivers – Overall population (N=275)

Explanatory variables*	Univariate Analyses			Multivariate Analysis		
	Number of patients	Least-Square (LS) Means [95% CI]	p value	Number of patients	Effect estimate [95% CI]	p value**
**Gender**			0.0562			
Female	88	72.93 [68.95 ; 76.90]				
Male	134	77.91 [74.69 ; 81.13]				
**Height SD compared to the general population**	222		<.0001	222	7.02 [4.30 ; 9.73]	<.0001
**Indication group**			0.0999			
GHD	138	77.57 [74.39 ; 80.76]				
Other condition	84	73.24 [69.16 ; 77.32]				
**Duration of hGH treatment (years)**	222		0.0023			
**Daily dose of rhGH** (kg/mg/day)	221		0.0065			

* Only variables with p value <0.2 are presented for univariate analyses

** Only variables with p value <0.05 are presented for multivariate analysis
